# Impact ionization and intervalley electron scattering in InSb and InAs induced by a single terahertz pulse

**DOI:** 10.1038/s41598-020-67541-1

**Published:** 2020-06-29

**Authors:** Steponas Ašmontas, Skaidra Bumelienė, Jonas Gradauskas, Romas Raguotis, Algirdas Sužiedėlis

**Affiliations:** 1 Center for Physical Sciences and Technology, 3 Saulėtekio Av., 10257 Vilnius, Lithuania; 2 Vilnius Gediminas Technical University, 11 Saulėtekio av., 10223 Vilnius, Lithuania; 3 Center for Physical Sciences and Technology, Saulėtekio av. 3, 10257 Vilnius, Lithuania

**Keywords:** Electronic properties and materials, Semiconductors, Materials for devices, Electronics, photonics and device physics

## Abstract

Electronic properties of InSb and InAs are sensitive to electric field due to their narrow forbidden energy gaps and big difference in effective masses of electrons in different conduction band valleys. Here we report impact ionization processes and redistribution of electrons between the Γ, L and X valleys induced by a single ultrashort terahertz (THz) pulse at 80 K temperature. Monte Carlo simulation revealed that electron motion in this case has near ballistic character. The threshold electric field of impact ionization increases as the THz pulse gets shorter, and the process of impact ionization essentially raises cooling rate of hot electrons. The L valley gets mainly occupied by electrons in InSb while the X valley holds the majority of electrons in InAs at strong electric fields, respectively above 20 kV/cm and 90 kV/cm. The calculated results are in good agreement with the available experimental data.

## Introduction

Successful development of terahertz radiation sources^1–6^ has stimulated intensive research on interaction of ultrashort laser pulses with matter^7–21^. When a semiconductor is illuminated with intense THz radiation, the average electron energy increases and the impact ionization process may begin^22^. The threshold energy of impact ionization *ε*_*th*_, in general, depends on semiconductor forbidden energy gap and electron mobility. Indium antimonide and indium arsenide are the narrow-gap semiconductors and therefore they have comparatively low value of the threshold energy. In n-InSb, 40 ns-long THz radiation pulse-induced impact ionization has been first experimentally investigated at liquid nitrogen temperature^23–25^. It was shown that the threshold electric field of impact ionization *E*_*th*_ grows with radiation frequency. Monte Carlo calculations revealed that *E*_*th*_ growth with frequency is related to the inertia of electron heating^26^. Z-scan technique has been employed to investigate nonlinear THz pulse transmission through InSb samples^7^. The observed nonlinear absorption and self-phase modulation of THz pulses were explained by ultrafast impact ionization processes driven by strong electric field. A sevenfold increase of electron density above the equilibrium was observed in n-type InSb under the action of 1 ps-long pulses with electric field strength up to 100 kV/cm^8^. THz-pump/THz-probe technique was applied to measure time-resolved absorption of laser radiation.

Later, the intervalley scattering of electrons and impact ionization in InAs were investigated at room temperature under the excitation of intense single-cycle THz 150 fs-long pulses^14,17^. Reflection geometry technique, typically demonstrating high sensitivity in bulk materials, enabled to reveal the impact ionization process to be started when THz electric field reached 110 kV/cm. No generation of electron–hole pairs was detected at weaker electric fields. Monte Carlo calculation showed that at room temperature *E*_*th*_ was of the order of 80 kV/cm when 150 fs-long pulses were used^27^. On the other hand, it was shown that at low temperature, *T* = 6 K, the threshold electric field of impact ionization in InAs was of the order of 10 kV/cm when nanosecond-long THz pulses were used^28^.

In this paper we present the results of Monte Carlo simulation in case when intense ultrashort, subpicosecond-long, THz pulse interacts with narrow-gap InSb and InAs semiconductors at *T* = 80 K. We study impact ionization processes and corresponding hot electron dynamics, population of electrons in the Γ and higher L and X valleys and sequence of their transfer between the valleys under the action of ultrashort THz pulse.

## Methods

Standard Monte Carlo simulation^29^ was employed to investigate electron dynamics and impact ionization processes in InSb and InAs. The model of three non-parabolic Γ, L and X valleys of the conduction band was chosen. Electron properties of bulk materials were directly related to the scattering mechanisms and to the band structure. Electron scattering mechanisms included in the model were those by non-elastic optical, intervalley, acoustical phonons and impurity scattering. The latter was treated by the third body exclusion method which encompasses the Brooks-Herring’s and Conwell-Weisskopf’s approximations^30^. The impact ionization probability per unit time was calculated as^31^

1 $$\begin{aligned} \lambda \left( \varepsilon \right) &= \frac{{2e^{4} }}{{\left( {4\pi \chi \chi _{0} } \right)^{2} \pi \hbar ^{3} }}\;\frac{{m_{e}^{2} }}{{m_{h} }}\left( {1 + \frac{{m_{h} }}{{m_{0} }}} \right)\frac{1}{{\varepsilon _{g} \phi \left( \varepsilon \right)}} \hfill \\ & \quad \times \int\limits_{0}^{{\varepsilon - \varepsilon _{g} }} {\left[ {\left( {\frac{{\omega _{2} }}{{2\sqrt {\varepsilon \varepsilon ^{\prime} } }} + \frac{{\alpha \omega _{1} \sqrt {\varepsilon \varepsilon ^{\prime} } }}{{\omega _{2} }}} \right)^{2} \times \ln \frac{{\omega _{1} + \omega _{2} }}{{\omega _{1} - \omega _{2} }} - 2\alpha \omega _{2} - 2\alpha ^{2} \varepsilon \varepsilon ^{\prime} \frac{{\omega _{1} }}{{\omega _{2} }}} \right]} \hfill \\ & \quad \times \phi \left( {\varepsilon - \varepsilon _{g} - \varepsilon ^{\prime} } \right)d\varepsilon ^{\prime} ,\hfill \\ \end{aligned}$$

where $\omega_{1} = \varepsilon \left( {1 + \alpha \varepsilon } \right) + \varepsilon ^{\prime} \left( {1 + \alpha \varepsilon ^{\prime} } \right)$, $\omega_{2} = 2\sqrt {\varepsilon \left( {1 + \alpha \varepsilon } \right)\varepsilon ^{\prime} \left( {1 + \alpha \varepsilon ^{\prime} } \right)}$, $\phi \left( \varepsilon \right) = \sqrt {\varepsilon \left( {1 + \alpha \varepsilon } \right)} \left( {1 + 2\alpha \varepsilon } \right)$. Here *α* is the parameter of non-parabolicity, *ε* and $\varepsilon ^{\prime}$ are the electron energies before and after scattering, respectively, *m*_*e*_ and *m*_*h*_ are the effective masses of an electron and hole, *m*_0_ is the free electron mass, *χ*_0_ is the permittivity of free space, and *χ* is the low frequency dielectric constant of a semiconductor. The parameters of InSb and InAs used for the calculations were respectively taken from the literature^32,33^.

Electron action in the **k**-space was simulated by the motion of *N* electrons’ ensemble according to the usual Monte Carlo method^34^. The angular probability distribution of the angle Θ between the wave vectors ${\mathbf{k}}$ and ${\mathbf{k^{\prime}}}$, before and after electron scattering, respectively, was taken as

2 $$P\left( \Theta \right) = \sin \Theta {\kern 1pt} {\kern 1pt} \left[ {\left( {{\mathbf{k}} - {\mathbf{k}}^{\prime }} \right)^{2} + \gamma ^{2} } \right]^{{ - 2}} ,$$

where *γ* is the reciprocal Debye length^35^. The states of secondary electrons after the impact ionization were recorded. Then, the simulation with each next secondary electron was done by one particle method remembering the recorded states of the third electron, and so forth.

## Results and discussion

**Figure 1:**
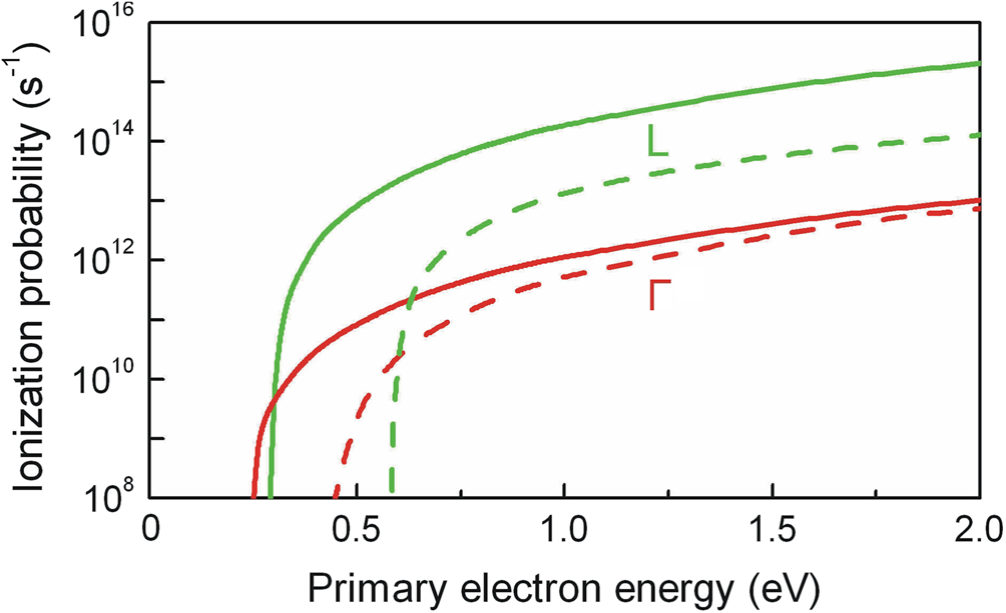
Electron impact ionization probabilities of InSb (solid lines) and InAs (dashed lines) in the respective Γ (red) and L (green) valleys versus primary electron energy at 80 K.

**Figure 2:**
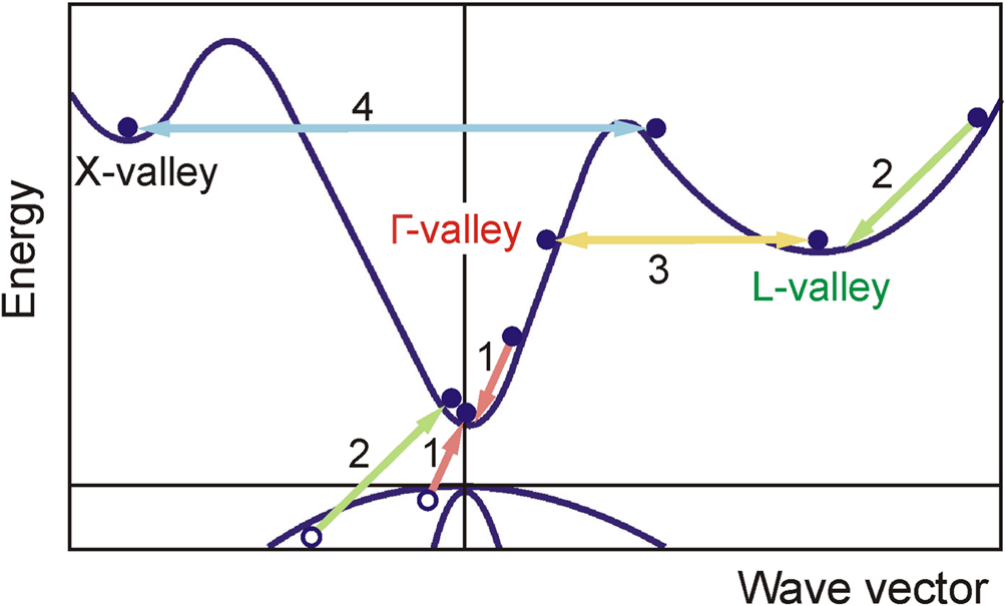
Illustration of dynamic mechanisms in InSb and InAs band structure: impact ionization in the Γ valley (1), impact ionization in the L valley (2), Γ-L intervalley scattering (3), and X-L intervalley scattering (4).

The impact ionization probability per unit time *λ*(*ε*) has similar dependence on the primary electron energy for both semiconductors (Fig. 1). The probability is significantly higher in the L valley than in the Γ valley for the high energy electrons. This difference is related to larger effective electron mass in the L valley (see Fig. 2; it also shows possible carrier transitions discussed below).

According to the authors^3^, intense single-cycle THz pulse is particularly suitable instrument to study directly interaction of strong electric field with matter within ultrashort timescales.

**Figure 3:**
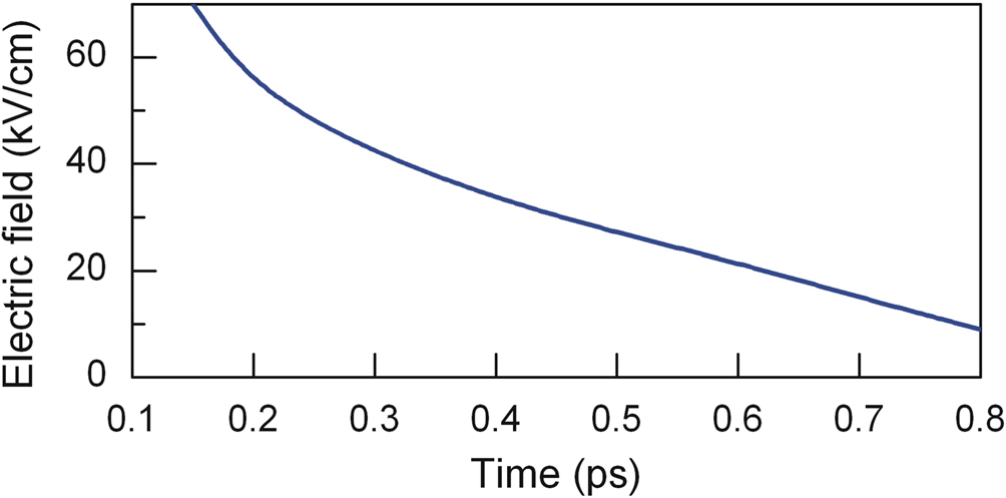
Dependence of the threshold electric field of impact ionization on pulse duration in n-InSb at 80 K.

For a single-cycle 0.8 ps-long pulse, the calculated threshold electric field of impact ionization in n-InSb was equal to 8.5 kV/cm at 80 K. The value of *E*_*th*_ increases as the pulse gets shorter (Fig. 3); simultaneously, the impact ionization rate decreases. The threshold electric field reaches 70 kV/cm at the pulse duration *τ* = 150 fs.

**Figure 4:**
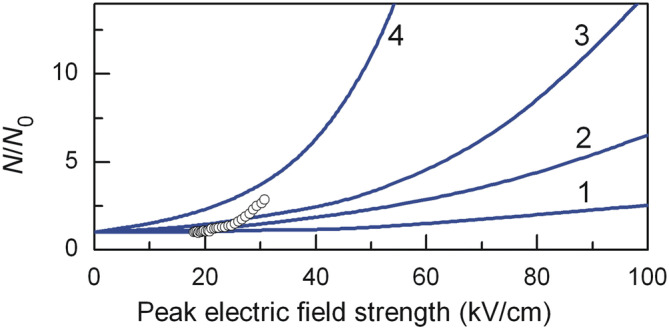
Dependence of normalized electron density on peak electric field after the THz pulse in InSb at 80 K. Pulse duration, ps: 1– 0.25, 2– 0.5, 3– 0.8, 4– 2. Solid lines represent theoretical calculation, circles are experimental data^7^.

As the applied electric field peak value increases, electron density after the THz pulse increases as well (Fig. 4). The number of generated electron–hole pairs grows very slowly in the case of ultrashort (*τ* = 0.25 ps) pulses, but this tendency gets sharper with longer pulses. For comparison, the experimental data of the normalized electron density measured under the action of 0.3 ps-long THz pulse^7^ are also presented in Fig. 4. Quite good qualitative agreement between both results can be seen. Sharper growth of the experimental results above 25 kV/cm peak field values most probably is due to total free carrier density measured during multiple reflections of the pulse within the sample^32^.

**Figure 5:**
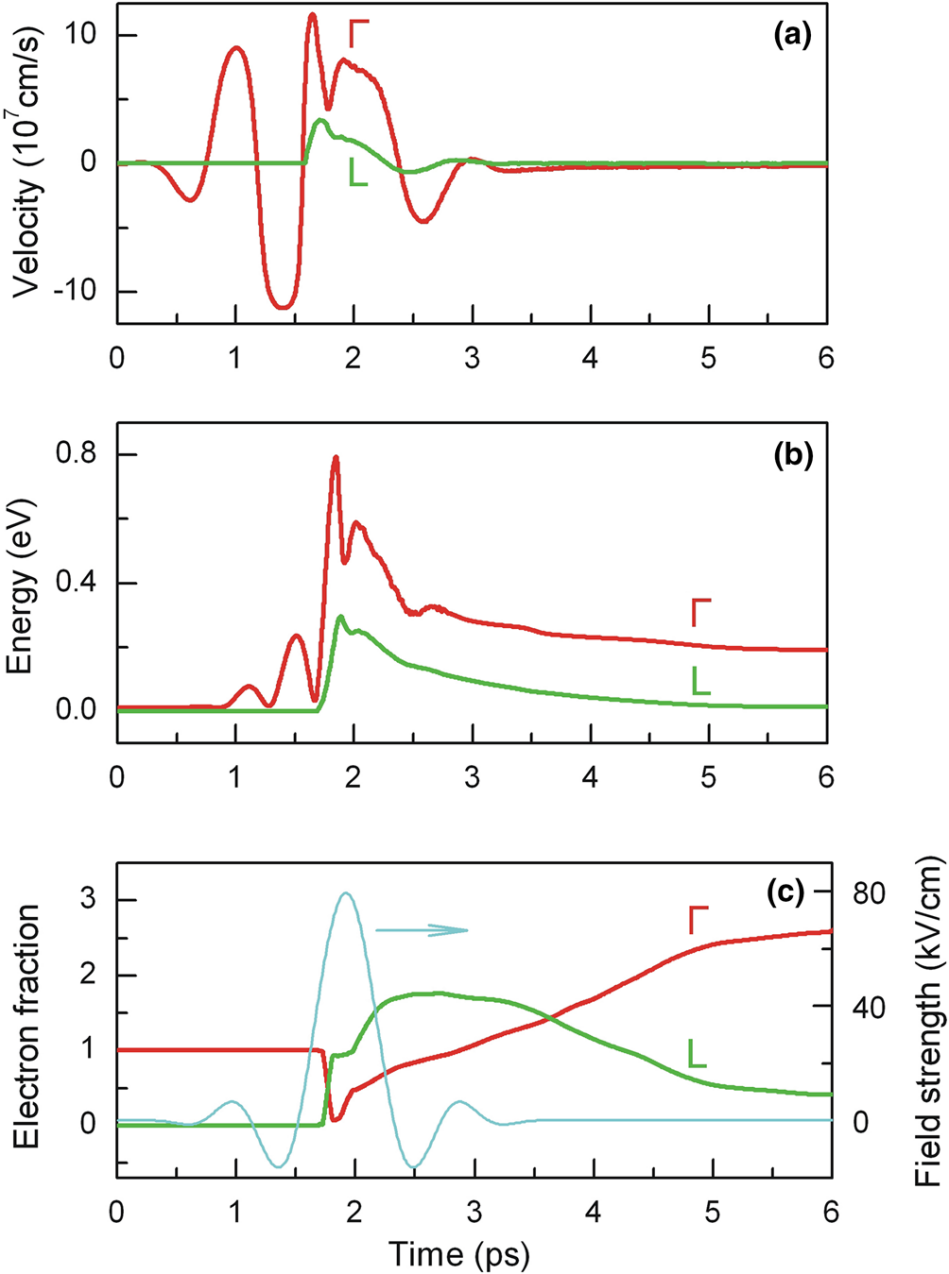
Electron dynamics in the Γ (red) and L (green) valleys of InSb with initial electron density *N*_0_ = 10^15^ cm^−3^ at 80 K: (**a**) Electron drift velocity under the action of electric field pulse; (**b**) Electron mean energy; (**c**) Changes of electron population with time and temporal shape of the single-cycle 0.8 ps-long electric field pulse (cyan).

The results of simulation of electron drift velocity *v*_*d*_ in InSb at 80 K under the action of electric field are shown in Fig. 5a. It is seen that the drift velocity follows electric field strength, and the maximum of *v*_*d*_, 1.2  ×  10^8^ cm/s, significantly exceeds its steady-state value of 4  ×  10^7^ cm/s^31^. Near ballistic motion of electrons at strong alternating electric field stipulates fast oscillation of their mean energy at the leading edge of the pulse (Fig. 5b).

Essential rise of electron mean energy in the Γ valley is observed during the action of strong electric field. Then hot electrons of the Γ valley having energy *ε* > 0.65 eV are scattered into the L valley^36^ at high rate (process 3 in Fig. 2). Therefore the population of electrons in the Γ valley drastically decreases (see Fig. 5c). Characteristic time of electron transfer between the Γ and L valleys in the strong electric field is found to be of the order of 50 fs, what is in good agreement with the experimentally measured Γ-to-L intervalley scattering time of 44 fs^37^.

Hot electrons having energy *ε* > *ε*_*th*_ initiate the impact ionization and generation of electron–hole pairs (processes 1 and 2). As a result, population of the Γ valley increases very rapidly since secondary electrons get into the Γ valley after the impact ionization. Since energy of the secondary electrons is low, then mean energy of the Γ valley electrons starts decreasing sharply in spite of existing strong electric field (see Fig. 5b). At the same time the electron drift velocity in the Γ valley also decreases (Fig. 5a). Later, the secondary electrons in the Γ valley get heated by still strong electric field and finally they are scattered into the L valley (process 3). As a result, the population of the L valley increases. Since the probability of impact ionization of the L valley electrons is sufficiently high (see Fig. 1), these electrons with *ε* > *ε*_*th*_ very quickly lose their energy due to the impact ionization (process 2), and their mean energy does not exceed *ε*_*th*_even at very strong electric field. This is the reason why the transition of hot electrons to the higher X valley is not observed in InSb. The calculation shows that impact ionization process is a dominant energy loss mechanism for the hot electrons with energy higher than *ε*_*th*_. The rise of population in the L valley ends after 600 fs (see Fig. 5c), and electrons from the L valley start returning back to the Γ valley; their mean energy decreases due to impact ionization process and electron scattering by intervalley and polar optical phonons. When these processes stabilize, the total almost threefold increase of electron density is observed.

**Figure 6:**
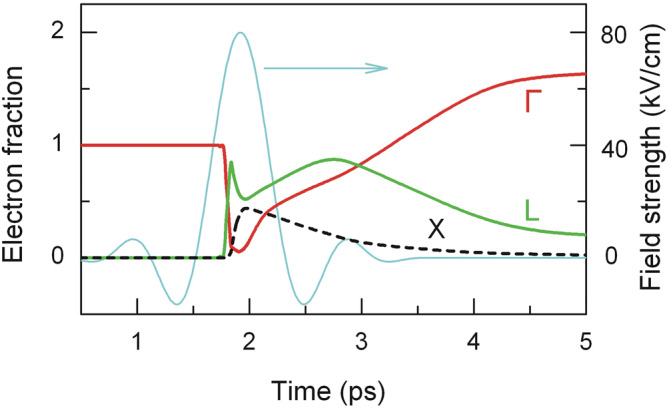
Dynamics of electron population in the Γ (red), L (green) and X (black) valleys in InAs with initial electron density *N*_0_ = 10^15^ cm^−3^ at 80 K. Cyan line represents single-cycle 0.8 ps-long electric field pulse.

A little different carrier dynamics is observed in InAs (Fig. 6). Sharp growth of mean energy of the Γ-valley electrons during the action of strong electric field also initiates hot electron (i.e., those having energy *ε* > 0.73 eV) scattering into the L valley (process 3). Owing to this, population of electrons in the Γ valley drops drastically, and the number of electrons in the L valley sharply increases. The calculated characteristic time of electron transfer between the Γ and L valleys in the strong electric field is of the order of 60 fs. Later, the L-valley electrons are further heated by still strong electric field and their mean energy increases. Electrons with energy *ε* > 0.29 eV are scattered from the L to the higher X valley (process 4). As a result, population of electrons in the L valley drops down and, consequently, it increases in the X valley (see Fig. 6).

The growth of electron number in the X valley lasts for about 100 fs, i.e., until the electric field is strong enough to heat electrons in the L valley. Later on, electrons from the X valley are scattered back to the L valley because their mean energy decreases in both valleys with diminishing electric field. Thus, population of electrons in the L valley increases again until the concentration of electrons in the X valley decreases considerably. Then electrons from the L valley get back to the Γ valley as the electric field vanishes, and the mean electron energy decreases due to their scattering by intervalley and optical phonons. The calculation shows that electron–hole pairs in InAs are mainly generated by the hot electrons of the Γ valley as the mean energy of electrons in the L and X valleys is lower than the threshold energy of impact ionization. Electron density increases by about 90% after 3 ps from the peak of the pulse.

**Figure 7:**
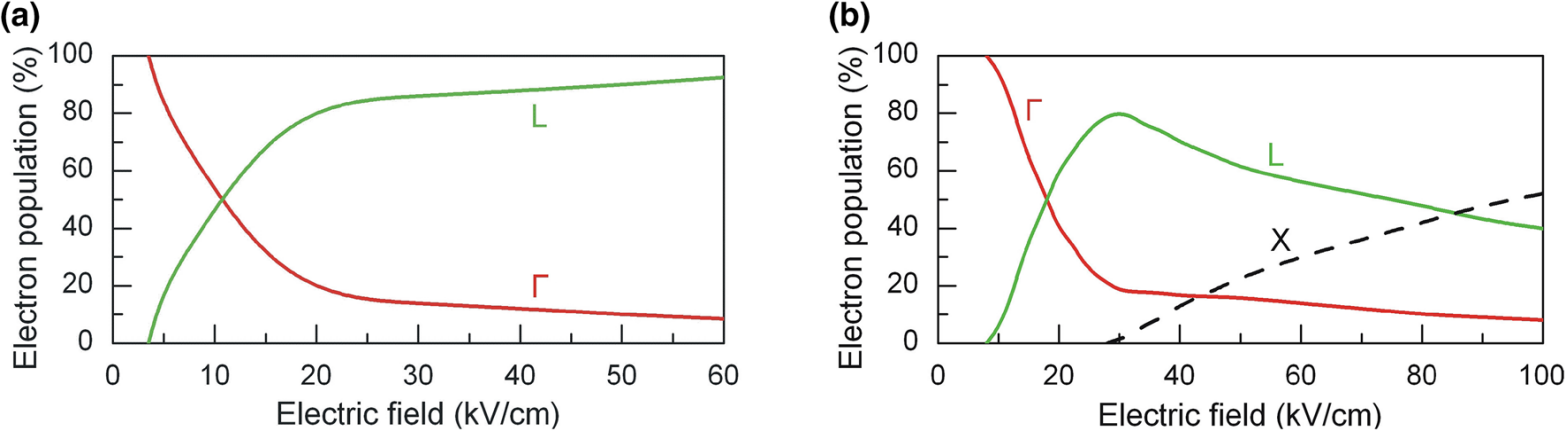
Relative population of electrons in the Γ (red), L (green) and X (black) valleys versus electric field peak value in: InSb (**a**) and InAs (**b**) with initial electron density *N*_0_ = 10^15^ cm^−3^ at 80 K.

Relative population of electrons in the valleys as a function of electric field strength both for InSb and InAs with initial electron density *N*_0_ = 10^15^ cm^−3^ is depicted in Fig. 7. In indium antimonide, redistribution of electrons during the action of intense ultrashort 0.8 ps-long pulse takes place mainly between the Γ and L valleys since electron mean energy in these valleys is not sufficient for electron transfer to the higher X valley (see Fig. 5b). As it was mentioned above, high probability of impact ionization of hot electrons in the Γ and L valleys does not allow electrons to reach energy sufficient for them to be scattered into the X valley. The electron transfer into the L valley starts at 3.5 kV/cm. More electrons are found in the L valley than in the Γ valley as the field exceeds 11 kV/cm.

In indium arsenide, electrons are scattered between the three, Γ, L and X, valleys as the electric field peak value rises (Fig. 7b). Transfer of electrons from the Γ into the L valley begins at 9 kV/cm-strong electric field. Population of electrons in the L valley increases rapidly with the electric field, and, starting from 18 kV/cm, more electrons are found in the L valley than in the Γ one. Transfer of electrons from the L valley into the X valley begins at 28 kV/cm, and the population in the L valley drops down. Finally, when the field exceeds 85 kV/cm, more electrons populate the X valley than the L one.

## Conclusions

The presented Monte Carlo simulation shows sharp growth of electron mean energy in the lower Γ valley during the action of intense THz radiation pulse. In InAs, the electrons being heated by the radiation electric field jump at first from the Γ valley into the L valley and then they are scattered up from the L valley to the X valley, whereas in InSb the electron distribution takes place mainly between the Γ and L valleys. Fast growth of the number of generated carriers with electric field exceeding its threshold value of impact ionization in InSb is caused by intense impact ionization of electrons of both L and Γ valleys. In case of InAs, generation of electron–hole pairs takes place mainly by means of the Γ-valley electrons since the mean energy of hot electrons in the L and X valleys appears to be lower than the threshold energy of the impact ionization.
